# Constraints on ore vectoring from geochemical fingerprints of porphyry style pyrite

**DOI:** 10.1038/s41598-025-99918-5

**Published:** 2025-05-28

**Authors:** Beata Naglik, Artur Sosnal, Magdalena Dumańska-Słowik, Tomasz Toboła, Dimitrina Dimitrova, Ryszard Habryn, Paweł Derkowski, Zbigniew Czupyt, Maciej Woszczyna, Marek Markowiak, Jaroslav Pršek

**Affiliations:** 1 Polish Geological Institute-National Research Institute, Upper Silesian Branch, 1 Królowej Jadwigi Str, 41-200 Sosnowiec, Poland; 2 Faculty of Geology, Geophysics, and Environmental Protection, AGH University of Krakow, 30 Mickiewicz Av, 30-059 Krakow, Poland; 3 Geological Institute, Bulgarian Academy of Sciences, 24 Acad. Georgi Bonchev Str, 1113 Sofia, Bulgaria; 4 Polish Geological Institute-National Research Institute, 4 Rakowiecka Str, 00-975 Warsaw, Poland

**Keywords:** LA-ICP-MS, Porphyry deposits, SHRIMP, Vectoring tools, Elemental ratios, Economic geology, Mineralogy

## Abstract

The sulfur isotope compositions of three generations of pyrite originated from skarns, stockwork, and late-stage, post-hydrothermal veins from three various zones of the porphyry style Myszków Mo–Cu–W deposit (center, circum-deposit, and periphery) were investigated as a proxy for the mineralized core of porphyry system. Overall, the mode of δ^34^S_pyrite_ decreases with time, from skarn- through main- up to late-stage of ore mineralization (with average values of + 6.13, + 5.65, and + 3.34 ‰, respectively). The gradual decrease in δ^34^S values outwards from the deposit core (av. 3.95 ‰), through circum-deposit (av. + 3.40‰) to distal zone (av. + 3.05 ‰) was detected only for late-stage pyrite. Both the temporal and lateral zonation of δ^34^S_pyrite_ could be explained by the progressive temperature decrease of the mineralized system and the mixing of ore-forming solutions with more dilute meteoric waters. The trace geochemistry of late-stage pyrite shows relatively constant values of Tl (from 0.13 to 0.14 ppm), Ti (9.10–10.30 ppm), Cr (9.94–12.37 ppm), and Mn (6.94–7.59 ppm) regardless of the zone of the Myszków Mo–Cu–W deposit. While, As (24.96–184.80 ppm), Sb (0.50–13.52 ppm), Bi (0.57–1.54 ppm) in pyrite and Sb/Te (0.06–1.62), Co/Bi (3.32–34.23), and Ag/Ni (0.006–0.140) increase with the proximity to the ore, contrary to Ag/Co which rises towards the periphery of the deposit (0.04–0.13). Ultimately, these results indicate that sulfur isotope data supported by trace geochemistry of late-stage pyrite can be potentially used as vectoring proxies to predict the likely direction to the mineralized center of a porphyry system.

## Introduction

Nowadays, sufficient supplies of mineral raw materials for rapidly developing green technologies have become one of the main economic challenges for modern industry. To meet the demand for strategic metals (e.g. copper, tungsten), new methods are needed to discover concealed, deep-seated (usually emplaced several kilometers below the Earth’s surface) and often small-size deposits. Drilling at such depths is extremely costly, so developing new pathfinders to target high-grade mineralization at reduced environmental and economic risk is of significant interest nowadays.

The most important world’s primary source of mineral raw materials such as Cu and Mo as well as important contents of critical and precious metals such as Au, Ag, W, and Re are porphyry Cu deposits and related epithermal Au deposits. Besides, sulfides extracted from porphyry-style mineralization might be a hosts for a number of metals critical for the global energy transition, including those which lack their own primary ores, e.g. Se and Te. They are intrusion-centered ore bodies, in which metal-bearing minerals precipitated from hydrothermal fluids within an intrusive host and surrounding country rocks^1^. Porphyry ore deposits typically occur within an alteration halo with characteristic mineralogical and chemical zoning patterns, providing key mineral guides, e.g. chlorite, epidote, etc. for ore prospecting^1^.

Therefore, to assist porphyry exploration, significant efforts have been made to develop effective geochemical vectoring tools, mostly based on whole rock geochemistry, e.g. Hf concentration and the elemental ratios of Sr/Y, V/Sc, and Th/U^2–4^. Quite recently, an alternative approach has been proposed using the mineral chemistry of individual magmatic and hydrothermal phases to predict the likely direction and distance to mineralized centers, as well as the potential metal endowment. Those geochemical exploration tools include porphyry indicator minerals (PIMs; e.g. zircon, apatite, plagioclase, biotite, magnetite, tourmaline), and porphyry vectoring and fertility tools (PVFTs, e.g. chlorite, epidote, alunite)^5–8^. The PIMs are traditionally used for recognition of fertile belts of igneous intrusions and associated hydrothermal alteration zones, while PVFTs can help to detect the location of concealed ore bodies with high precision^7^. As being less prone to nugget effects, mineral indicators define a broader footprint and give directional information at greater distances than it can be obtained via bulk assay^9–11^. However, there are some limitations to applying these tools. To obtain perfect mineral indices all physicochemical conditions (the pressure, temperature, and oxygen fugacity) prevailing during the crystallization of minerals as well as the elemental partitioning between co-crystallizing phases should be controlled and considered carefully^12^.

In turn, the potential of using trace elements in pyrite for exploration has been recognized since the end of the 1960s and has been still evolving due to the advances in LA-ICP-MS. Some works have been done to use the geochemistry of pyrite as a vectoring tool towards mineralization center in hydrothermal ore systems, including the porphyry ones^13–16^. In the case of the porphyry Myszków Mo–Cu–W deposit, the analysis of temporal variations of pyrite geochemistry provided evidence of metallogenic processes, including the role of boiling as a key ore-forming mechanism^17^. However, the use of some trace elements in pyrite as an exploration tool has not be assessed here up to now.

As a result, further new indices are welcome to support the prospecting wherever bulk or mineral proxies are ineffective. Another way to detect the proximity to the mineralized porphyry systems could be isotope geochemistry as the stable isotopic alteration halo can be observed at distances of up to 3 km laterally around ore mineralization, or even more^18^. Stable isotopes of sulfur in hydrothermal sulfides and sulfates primarily provide information on the potential source(s) of sulfur and the nature of mineralizing fluids^19^. The fractionation of the sulfur isotopes might involve several mechanisms, for example: simple cooling, fluid mixing, contamination of biogenic sulfur, boiling, and inorganic sulfate reduction associated with oxygen fugacity (*f*O_2_) fluctuations^20–22^. Sometimes these mechanisms might produce characteristic zonation patterns, and therefore the idea of using sulfur isotope data as vectoring proxies for exploration under cover has already started to develop and seems to evolve shortly^20–22^. Lickfold^21^ documented the spatial δ^34^S_pyrite_ isotope zonation in porphyry copper–gold deposits from the Goonumbla districts (New South Wales, Australia), pointing to the overall increase in δ^34^S_pyrite_ values with increasing distance from the mineralization center. Similarly, Wilson et al.^22^ concluded that sulfides proximal to fluid sources tend to be isotopically lighter than in more distal samples, i.e. from propylitic halos. However, spatial zonation in δ^34^S_pyrite_ in porphyry-style sulfides is not so obvious in all porphyry systems worldwide. For example, the Silver Creek porphyry Mo deposit in Colorado (USA) shows no systematic spatial or temporal isotopic variation^23^.

Hence, this study was made to trace the possible geochemical variations of pyrite (δ^34^S_pyrite_, trace element distribution) studied systematically from the center of mineralization to the most distal parts of the hydrothermal mineralizing system of the porphyry Myszków Mo–Cu–W deposit (the Krakow-Lubliniec Fault Zone, Poland). Moreover, the potential of sulfur isotopic zonation as a vector to hidden mineralized targets was evaluated and its implications for porphyry ore genesis were provided. Specifically, we discussed (1) the isotope zoning of ore system, (2) the physicochemical factors affecting the mineral chemistry, (3) the mechanism of isotope fractionation under a high-temperature mineralization environment, (4) the metal isotopes’ behavior during hydrothermal alteration and ore formation processes, (5) the source(s) of metals endowment, and (6) the evolution of mineralizing fluids along with time.

## Geological settings

**Fig. 1:**
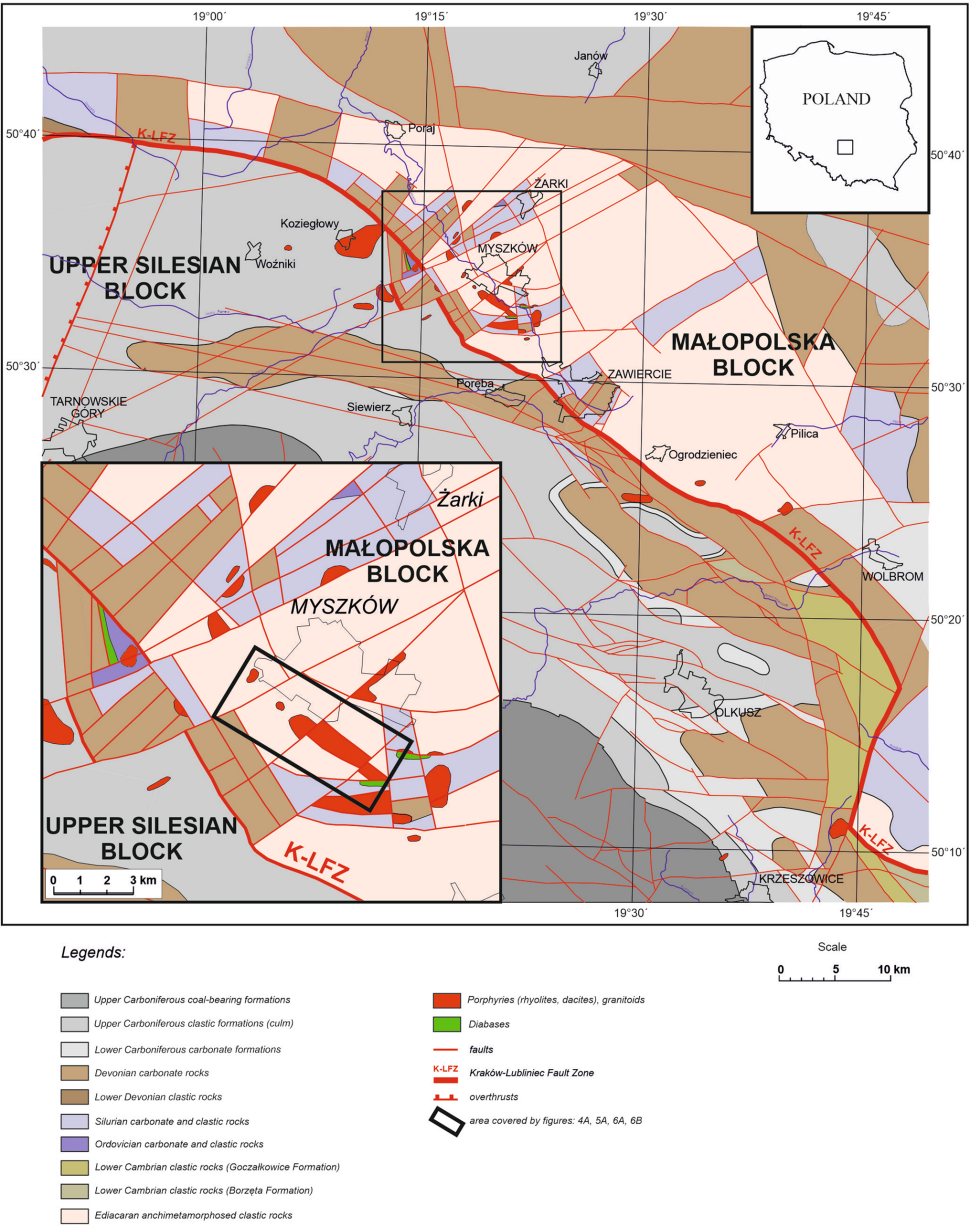
Geological map of the porphyry Mo–Cu–W Myszków deposit (modified after^30^).

The porphyry Myszków Mo–Cu–W deposit and the other prospects along the Kraków-Lubliniec Fault Zone (KLFZ, see Fig. 1) provide unusual examples of porphyry-style mineralization with anomalously high tungsten content, an unconventional geotectonic regime (transition from post-collisional to intra-plate settings; see^24–26^ and late Carboniferous to early Permian age of mineralization related to the Variscan continent–continent type collision between Baltica and Gondwana^27,28^. The KLFZ separates the two distinctive tectonic units: (1) the Upper Silesian Block, and (2) the Małopolska Block, both of which have their own distinctive history of evolution. The Upper Silesian Block constitutes a sector of the *Brunovistulicum* composite terrane, while the Małopolska Block is a thinned marginal part of the Baltica craton^25^. The Małopolska Block, which hosts the porphyry Myszków Mo–Cu–W deposit, is composed of two main structural units: (1) Neoproterozoic (Ediacaran) and Early Paleozoic basement and (2) Mesozoic cover. The Neoproterozoic basement of the Myszków area is composed of the anchimetamorphosed and tectonically deformed flysh-like silicoclastic rocks, which were intruded by a Late Carboniferous granitoid body associated with ore mineralization^25,29^.

Despite the different age of the Myszków Mo–Cu–W deposit, the overall geochemical characteristics, mineralogy, and vein morphology are typical of calc-alkaline porphyry copper deposits elsewhere (^29^ and the references therein). Some characteristic features of the Myszków Mo–Cu–W deposit that are not typical of most porphyry Cu deposits, particularly those in Europe, include much lower concentrations of Au and Ag, a higher concentration of W, and the presence of tungsten-bearing minerals in the ore-related pluton^29^.

The deposit has four distinct types of hypogene alterations, i.e. potassic, propylitic, argillic, and phyllic providing key guides for exploration in this area^31^. The ore mineralization resulted from both contact metamorphism (early, skarn-type mineralization) and post-magmatic, hydrothermal activity (main hydrothermal mineralization). The precipitation of ores occurred in a series of pulses controlled by different fissure systems and was characterized by diverse paragenetic sequences. Generally, three periods of mineralization have been defined at the Myszków deposit: (1) early, skarn-forming (period I), (2) main, hydrothermal (period II), and (3) late, post-ore (period III)^31,32^.

Period I is represented by magnetite-sulfide mineralization within hornfelses, skarns, and metasomatites. Magnetite, pyrite, hematite, and sphalerite were the principal minerals related to this stage of mineralization. Scheelite, pyrrhotite, galena, ilmenite, marcasite, cubanite, bismuthinite, native Bi, and Bi sulfosalts are found more rarely, while molybdenite could be found in skarns only sporadically. The oxide-sulfide mineral assemblage was formed under the temperature of 550–330 °C^31^.

Within period II, different stages of ore deposition have been distinguished: (1) feldspar-molybdenite veins with biotite, (2) quartz-feldspathic, pegmatitic veins, (3) quartz veins with molybdenite and scheelite (stockwork system), (4) black quartz veins with molybdenite, (5) quartz-polymetallic veins (without molybdenite). The stages differ in a suite of minerals due to the different physicochemical conditions of ore deposition. The order of phase crystallization was defined based on the cross-cutting sequence of veins^31^ and reflects decreasing temperature with time (800–160 °C).

Finally, period III produced mainly veins of ankerite, barite-carbonate-fluorite mineralization, and carbonate-galena-sphalerite assemblages^31^. Late-stage veins are widely distributed and occur with no spatial correlation with different types of hydrothermal alteration in the host rocks. Veins of this latter type are mineralized with pyrite, chalcopyrite, sphalerite, and galena^31^. They were probably formed at a low temperature under subsurface conditions^31,32^.

The porphyry Myszków Mo–Cu–W deposit was recognized as a result of an intensive drilling program (over 30 km of drilling) carried out in the years 1975–1992 covering an area of 0.54 km^2^ and down to the depth of 1300 m^29^. It remains the only European porphyry-style deposit of the Variscan age (297.5 + /− 0.5 Ma) still undergoing active exploration^29,34^. Molybdenum, Cu, and W occur as major valuable metals, while Ag, Au, and Re have the potential to be by-products. The first resource estimates of the Myszków deposit in 1993 allowed it to be classified as a Polish C2 resources category[^29^ and the references therein]. The recent data refer to the estimated resource of 726 Mt at 0.121 percent copper, 0.617 percent molybdenum, 0.0404 percent tungsten, and 2.2 g/t silver, based on a cutoff 0.085 percent molybdenum equivalent (^34^ and the references therein). Hence, the Myszków Mo–Cu–W porphyry deposit is considered one of the ten largest molybdenum deposits in the world in terms of tonnage^33^. It might be the first molybdenum-producing deposit in the European Union, and a supplementary source of tungsten, in addition to the currently active Mittersill mine in Austria and the Panasqueira mine in Portugal^33^.

## Characteristics of pyrite

**Fig. 2:**
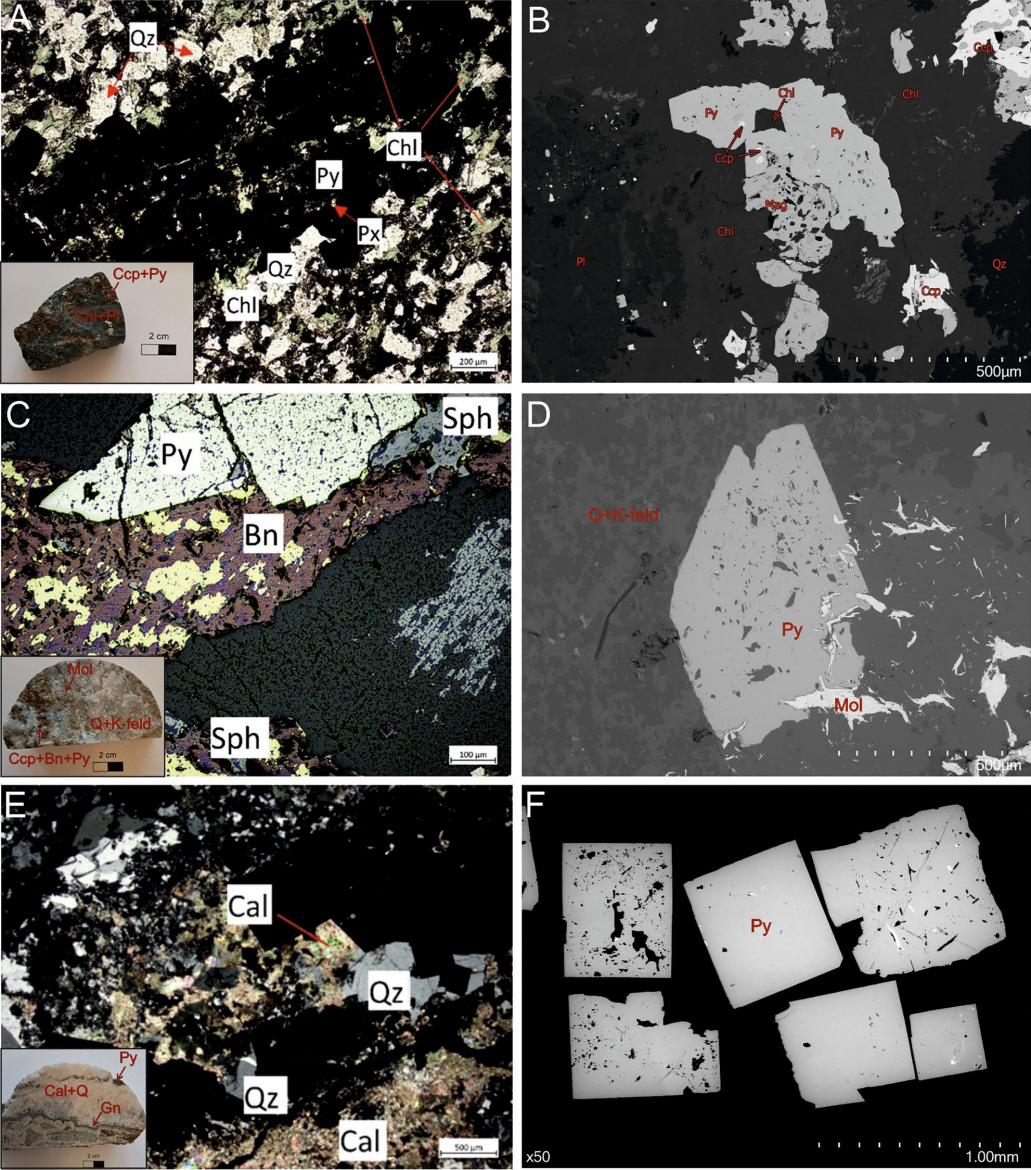
Macro- and micro-scale photos together with BSE images of pyrite crystals and their host rocks: (**A**, **B**)—skarn-hosted pyrite mineralization; (**C**, **D**)—pyrite from stockwork system (main-stage pyrite); (**E**, **F**)—late-stage pyrite. The dark spots are inclusions of gangue minerals and the light spots are mostly inclusions of galena.

Pyrite representing three different periods of ore mineralization in the porphyry Myszków Mo–Cu–W deposit has specific textural and geochemical characteristics. The early-stage pyrite is found in quartz-chlorite-biotite veins with ores such as magnetite, molybdenite, and scheelite. It also occurs in association with chalcopyrite as impregnations within metamorphic rocks, i.e. skarns, hornfelses, and metasomatites (Fig. 2A). The skarn-type pyrite forms anhedral grains with characteristic porous microtexture (Fig. 2B) and hosts abundant mineral inclusions of chlorite, magnetite, chalcopyrite and rarely bismuthinite and bastnäsite-(Ce)^35^. It is distinctly enriched in Co (up to 3966.11 ppm), Ni (up to 2552.62 ppm), Mn (up to 70.32 ppm), and Se (up to 103.94 ppm)^17^. The pyrite representing the main, hydrothermal stage of ore mineralization is found within the stockwork veins of quartz, K-feldspars, and calcite, with coexisting molybdenites, scheelite, chalcopyrite and locally bornite. It forms commonly anhedral grains (Fig. 2C,D) with a diversified set of inclusions of cassiterite, rutile, anatase, monazite-(Ce), xenotime, cerianite-(Ce) and others. The main stage pyrite was formed under gentle boiling to non-boiling conditions^35^. It is enriched in Co (up to 3312.65 ppm), Ni (up to 1469.09 ppm), Se (up to 184.24 ppm), and Te (up to 117.50 ppm)^17^. The late-stage pyrite occurs within the latest carbonate-quartz veins and druses. It forms euhedral grains (Fig. 2E,F) found in paragenesis with sphalerite, galena, chalcopyrite, fluorite, celestine, and sometimes REE-bearing phases.

## Results

### Zonation of δ^34^S_pyrite_: grain scale

**Fig. 3:**
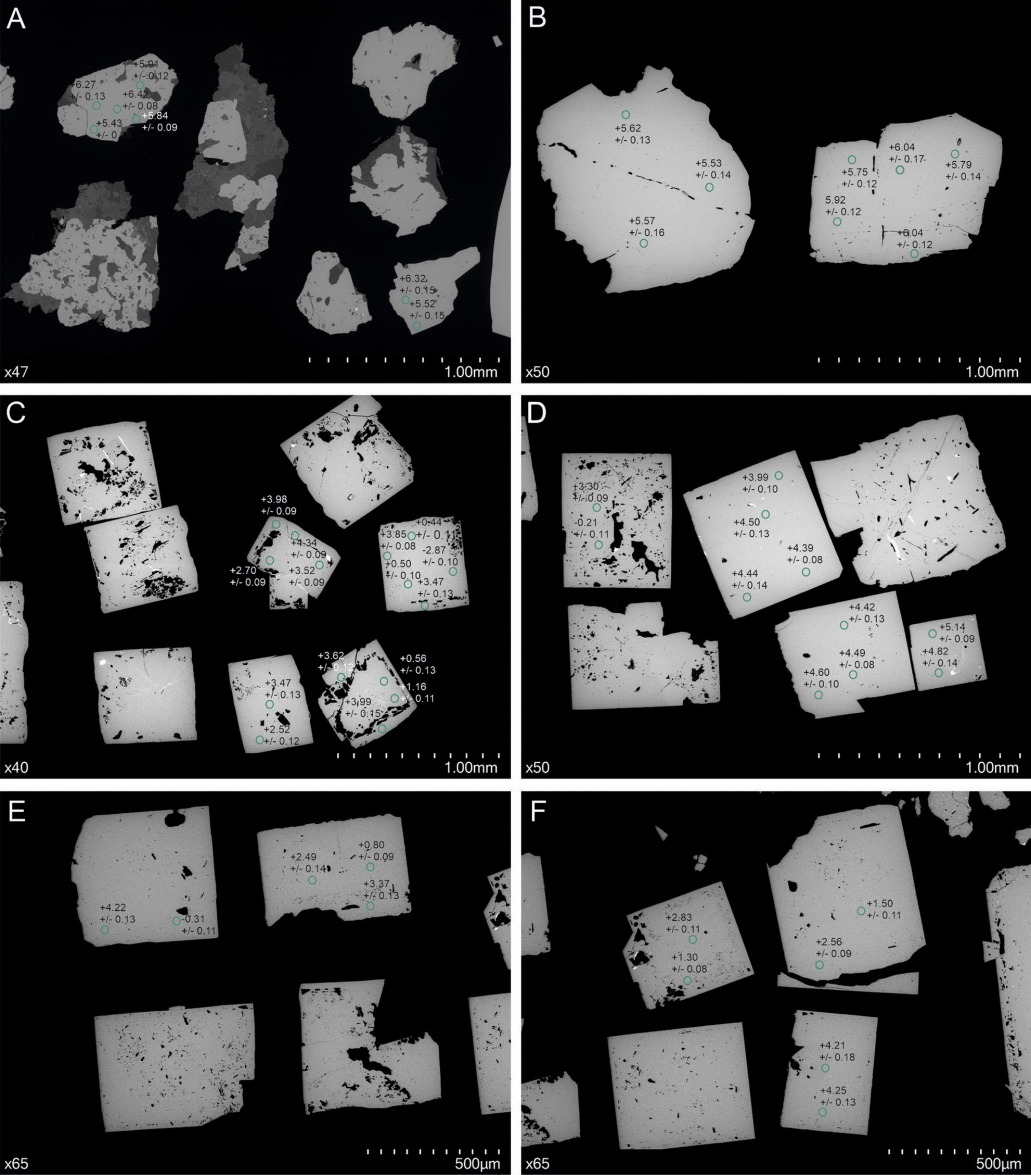
Differentiation of δ^34^S data at the pyrite grains scale: (**A**)—skarn-hosted pyrite mineralization (Pz-32; 446.1 m); (**B**)—pyrite from stockwork system (main-stage pyrite) (Pz-16; 352.2 m); (**C**)—inclusions-rich late-stage pyrite (Pz-10; 278.2 m); (**D**)—late-stage pyrite (Pz-20; 928.9 m); (**E**, **F**)—inclusions-rich late stage pyrite (Pz-24; 311.8 m).

Skarn-hosted and stockwork pyrite species show a rather homogenous distribution of δ^34^S within single grains (Fig. 3A,B). Inclusions-rich, late-stage pyrite displays microscale variations with heterogenous shifts toward negative δ^34^S values (Fig. 3C–F). Few late-stage pyrite grains exhibit core-rim textures, sometimes reflecting different δ^34^S composition (Fig. 3C). In contrast, specimens devoid of any inclusions, show rather homogenous composition of δ^34^S (Fig. 3D).

### Lateral and temporal zonation of δ^34^S_pyrite_: deposit scale

In general, the mode of δ^34^S_pyrite_ decreases with time from skarn- through the main- up to the late-stage of ore mineralization, with average values of + 6.13, + 5.65, and + 3.34 ‰, respectively (Table 1). Both skarn- and stockwork-type mineralization recorded positive results between the range of + 3.54 and + 10.50‰ with the average value at + 6.13 and + 5.65‰, respectively. The δ^34^S of late-stage pyrite shows a larger variation between −3.22 and + 7.19‰, with the main value found at + 3.34‰ (Table 1).

**Table 1:** The δ^34^S_pyrite_ composition across different paragenetic sequences.

Stage of mineralization	δ^34^S_pyrite_ (‰)			No. of points
	Min	Max	Average	
Skarn	+ 4.35	+ 10.50	+ 6.13	29
Main-hydrothermal (stockwork)	+ 3.54	+ 7.35	+ 5.65	160
Late, post-hydrothermal	− 3.22	+ 7.19	+ 3.34	103

**Fig. 4:**
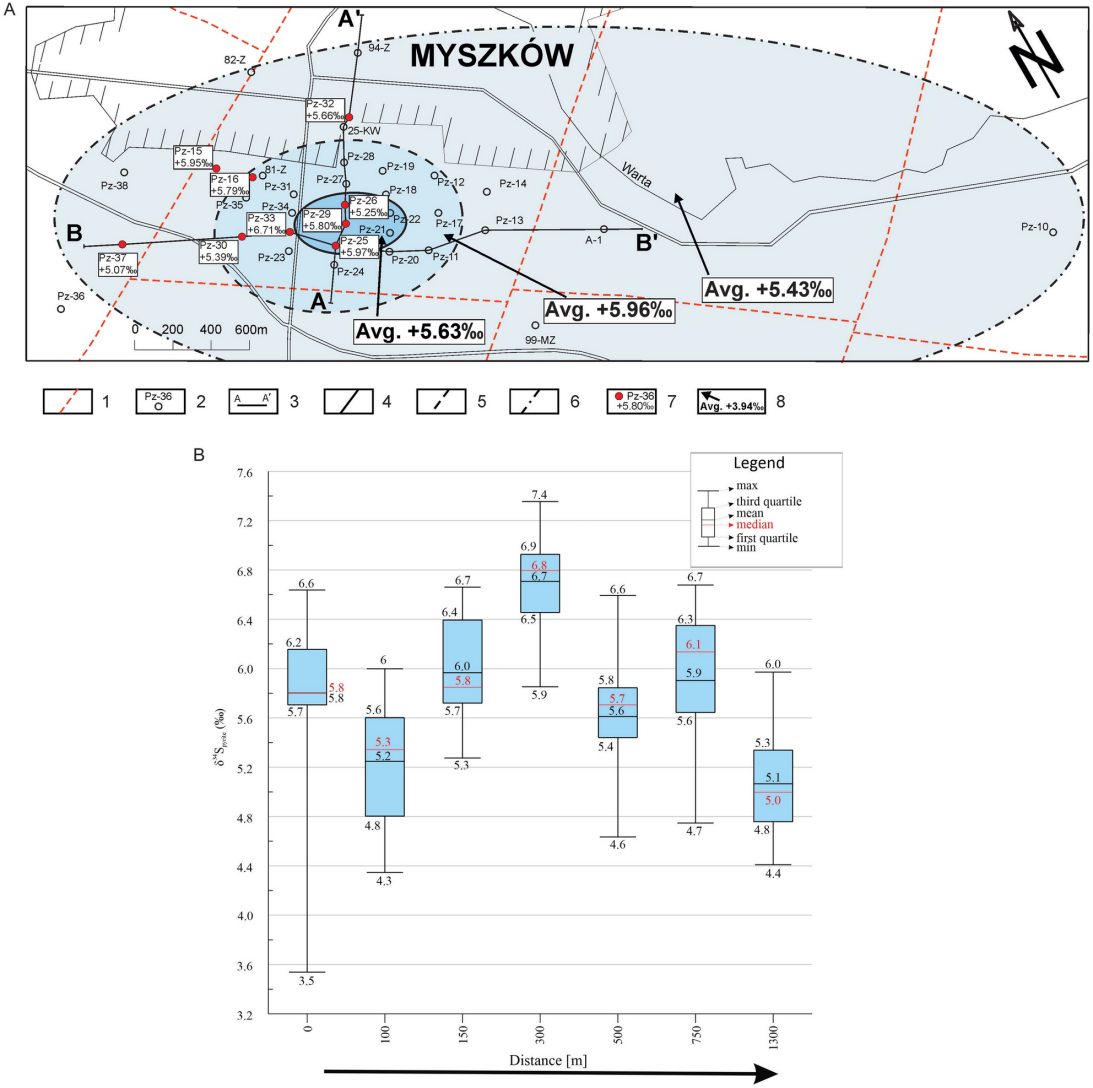
(**A**) Map of the Myszków Mo–Cu–W deposit with the δ^34^S composition of main-stage pyrite. 1—faults, 2—boreholes, 3—geological cross-section lines, 4—limit of deposit zone, 5—limit of circum-deposit zone, 6—limit of deposit’s peripheral zone, 7—investigated boreholes with δ^34^S anomaly, 8—averaged values of δ^34^S anomaly. (**B**) Boxplots of the δ^34^S data for main-stage pyrite. Boreholes are shown from the left (the core of the deposit) to the right (the periphery of the deposit); not in a scale.

The main-stage ore mineralization event is characterized by the homogenous distribution of δ^34^S_pyrite_ data. Neither lateral nor vertical pattern is observed (Table 2, Fig. 4A,B). The δ^34^S values of main-stage pyrite range from + 3.54‰ (sample Pz-29; 576.4 m) to + 7.35‰ (sample Pz-33; 415.8 m).

**Table 2:** The δ^34^S composition of main-stage pyrite as a function of distance-to-center.

Distance-to-center (m) (borehole; depth)	δ^34^S_pyrite_ (‰)			No. of points	Location	δ^34^S_pyrite_ (‰)
	Min	Max	Average/Std dev			Average
0 (Pz-29; 576.4)	+ 3.54	+ 6.64	+ 5.80/0.70	14	Deposit zone	+ 5.63
100 (Pz-26; 494.0)	+ 4.35	+ 6.00	+ 5.14/0.52	9		
100 (Pz-26; 518.3)	+ 4.72	+ 5.88	+ 5.36/0.38	9		
150 (Pz-25; 791.2)	+ 5.27	+ 6.66	+ 5.97/0.42	13		
300 (Pz-33; 415.8)	+ 5.85	+ 7.35	+ 6.71/0.40	17	Circum-deposit zone	+ 5.96
500 (Pz-30; 589.4)	+ 4.63	+ 6.59	+ 5.39/0.68	17		
500 (Pz-16; 352.2)	+ 5.53	+ 6.04	+ 5.79/0.15	17		
500 (Pz-32; 1059.5)	+ 5.22	+ 6.08	+ 5.66/0.19	18	Peripheral zone	+ 5.43
750 (Pz-15; 289.4)	+ 4.75	+ 6.68	+ 5.84/0.69	15		
750 (Pz-15; 387.9)	+ 6.32	+ 5.87	+ 6.07/0.18	4		
1300 (Pz-37; 754.5)	+ 4.41	+ 5.97	+ 5.07/0.36	15		
1300 (Pz-37; 833.9)	+ 4.41	+ 5.71	+ 4.87/0.34	16

**Fig. 5:**
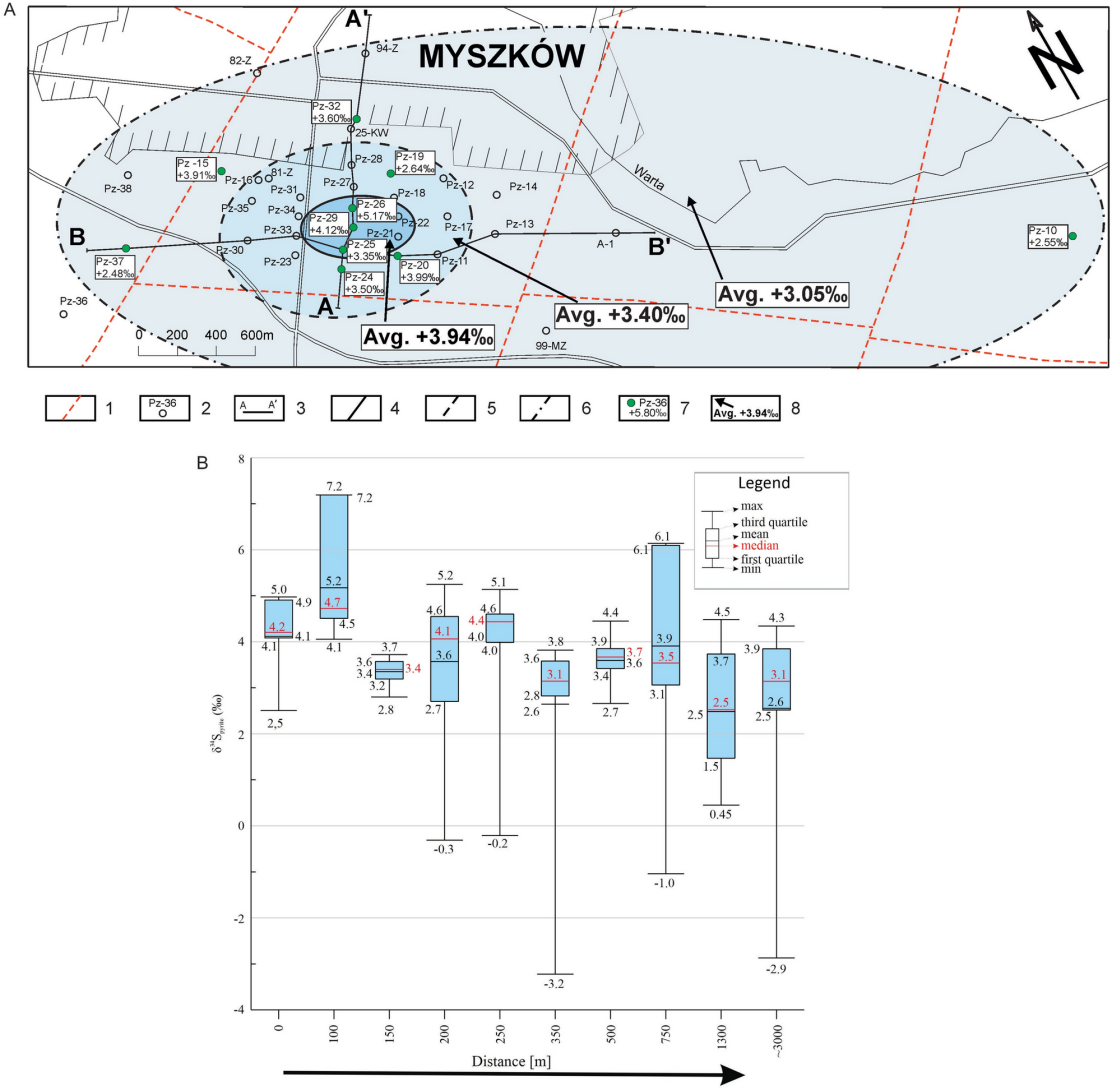
(**A**) Map of the Myszków Mo–Cu–W deposit with the δ^34^S composition of late-stage pyrite. 1—faults, 2—boreholes, 3—geological cross-section lines, 4—limit of deposit zone, 5—limit of circum-deposit zone, 6—limit of deposit peripheral zone, 7—investigated boreholes with δ^34^S anomaly, 8—averaged values of δ^34^S anomaly. (**B**) Boxplots of the δ^34^S data for late-stage pyrite. Boreholes are presented from the left (the core of the deposit) to the right (the periphery of the deposit); not in a scale.

**Fig. 6:**
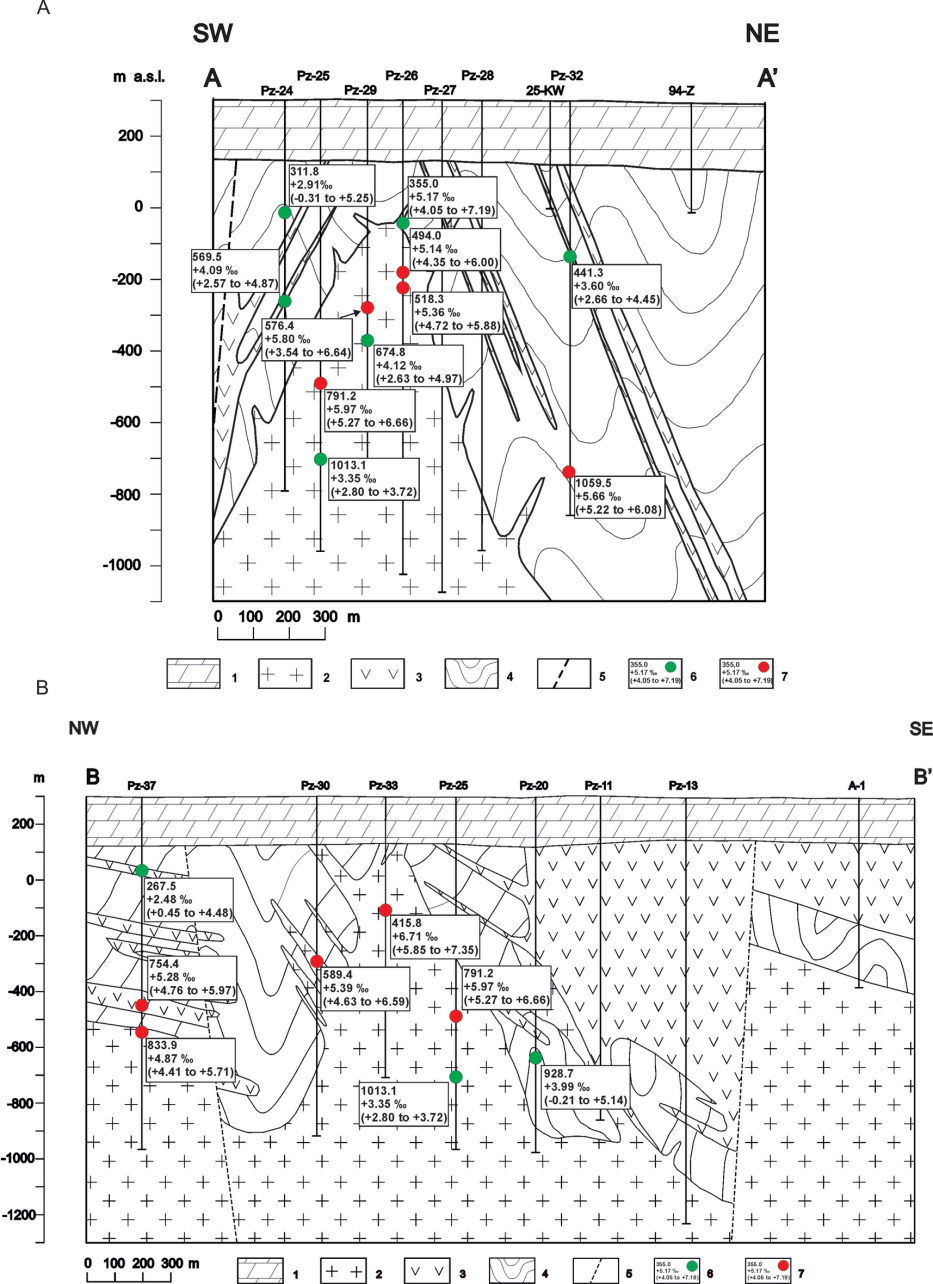
(**A**) SW-NE cross section through the Myszków deposit. 1—Triassic sediments, 2—granitoids, 3—dacitoids, 4—metamorphosed clastic rocks of Vendian-Early Cambrian, 5—faults, 6—investigated points with late-stage mineralization (depth, mean δ^34^S anomaly, minimum to maximum δ^34^S anomaly values), 7—investigated points with main stage mineralization (depth, mean δ^34^S anomaly, minimum to maximum δ^34^S anomaly values). (**B**) NW-SE cross-section through the Myszków deposit. 1—Triassic sediments, 2—granitoids, 3—dacitoids, 4—metamorphosed clastic rocks of Vendian-Early Cambrian, 5—faults, 6—investigated points with late-stage mineralization (depth, mean δ^34^S anomaly, minimum to maximum δ^34^S anomaly values), 7—investigated points with main stage mineralization (depth, mean δ^34^S anomaly, minimum to maximum δ^34^S anomaly values).

In turn, the decrease in δ^34^S_pyrite_ with increasing distance-to-center is observed for the late-stage mineralization event (Table 3, Figs. 5A,B and 6.). Isotopic zonation is reflected by a progressive δ^34^S_pyrite_ depletion outward from the central zone (+ 3.94‰) through the circum-deposit zone (+ 3.40‰) to the peripherical zone (+ 3.05‰). The δ^34^S values of late-stage pyrite range from −3.22‰ (sample Pz-19; 388.4 m) to + 7.19‰ (sample Pz-26; 355.0 m).

**Table 3:** The δ^34^S composition of late-stage pyrite as a function of distance-to-center.

Distance-to-center (m) (borehole; depth)	δ^34^S_pyrite_ (‰)			No. of points	Location	δ^34^S_pyrite_ (‰)
	Min	Max	Average/Std dev			Average
0 (Pz-29; 674.8)	+ 2.63	+ 4.97	+ 4.12/0.79	12	Deposit zone	+ 3.94
100 (Pz-26; 355.0)	+ 4.05	+ 7.19	+ 5.17/1.21	4		
150 (Pz-25; 1013.1)	+ 2.80	+ 3.72	+ 3.35/0.26	12		
200 (Pz-24; 311.8)	−0.31	+ 5.25	+ 2.91/1.54	15	Circum-deposit zone	+ 3.40
200 (Pz-24; 569.5)	+ 2.57	+ 4.87	+ 4.09/0.76	19		
250 (Pz-20; 928.7)	−0.21	+ 5.14	+ 3.99/1.40	11		
350 (Pz-19; 388.4)	−3.22	+ 3.82	+ 2.64/1.64	16		
500 (Pz-32; 441.3)	+ 2.66	+ 4.45	+ 3.60/0.37	19	Peripheral zone	+ 3.05
750 (Pz-15; 701.3)	−1.06	+ 6.14	+ 3.91/2.04	11		
1300 (Pz-37; 267.5)	+ 0.45	+ 4.48	+ 2.48/1.29	18		
~ 3000(Pz-10; 278.2)	−2.87	+ 4.34	+ 2.55/1.73	20

Moreover, the vertical pattern of δ^34^S_pyrite_ (progressive depletion in heavy sulfur toward the surface in the circum-deposit zone and peripherical zone; see Table 3) was also observed. Interestingly, within the intrusion itself the trend is opposite—the highest value of δ^34^S_pyrite_ was recorded at the shallowest level of the deposit (Table 3).

### Fluid inclusions

The various types of fluid inclusion assemblages (FIAs), found in quartz and calcite coexisting with the younger generation of pyrite, were distinguished, depending on their relationships to crystallographic axes and mode of occurrence^36–38^. They were characterized by the homogenization temperatures (Th) and salinity (Table 4) aiming to find possible drivers for δ^34^S_late-pyrite_ fractionation mechanisms.

**Table 4:** Microthermometric data for quartz and calcite coexisting with the late-stage pyrite.

Distance-to-center (m) (borehole; depth) Mineral (type of FIAs)	Homogenization temperatures [°C]			Salinity [wt%]		
	Min	Max	Average	Min	Max	Average
0 (Pz-29; 674.8)						
Quartz (primary FIAs)	119.5	343.1	185.1	1.2	5.1	2.3
Calcite (primary FIAs)	73.6	326.2	217.4	–	–	–
100 (Pz-26; 355.0)						
Quartz (primary FIAs)	44.5	194.3	136.0	1.2	2.7	2.3
Quartz (secondary FIAs)	221.4	265.5	237.0	4.2	6.0	5.1
200 (Pz-24; 311.8)						
Quartz (primary FIAs)	140.0	284.4	199.7	1.7	7.2	4.0
Quartz (secondary FIAs)	264.5	366.1	325.0	0.9	8.0	3.1
500 (Pz-32; 441.3)						
Quartz (primary FIAs)	147.4	215.3	180.5	–	–	–
Quartz (secondary FIAs)	247.0	328.3	281.9	2.7	4.7	3.7
Calcite (primary FIAs)	55.4	135.4	84.3	1.2	4.8	3.4

**Fig. 7:**
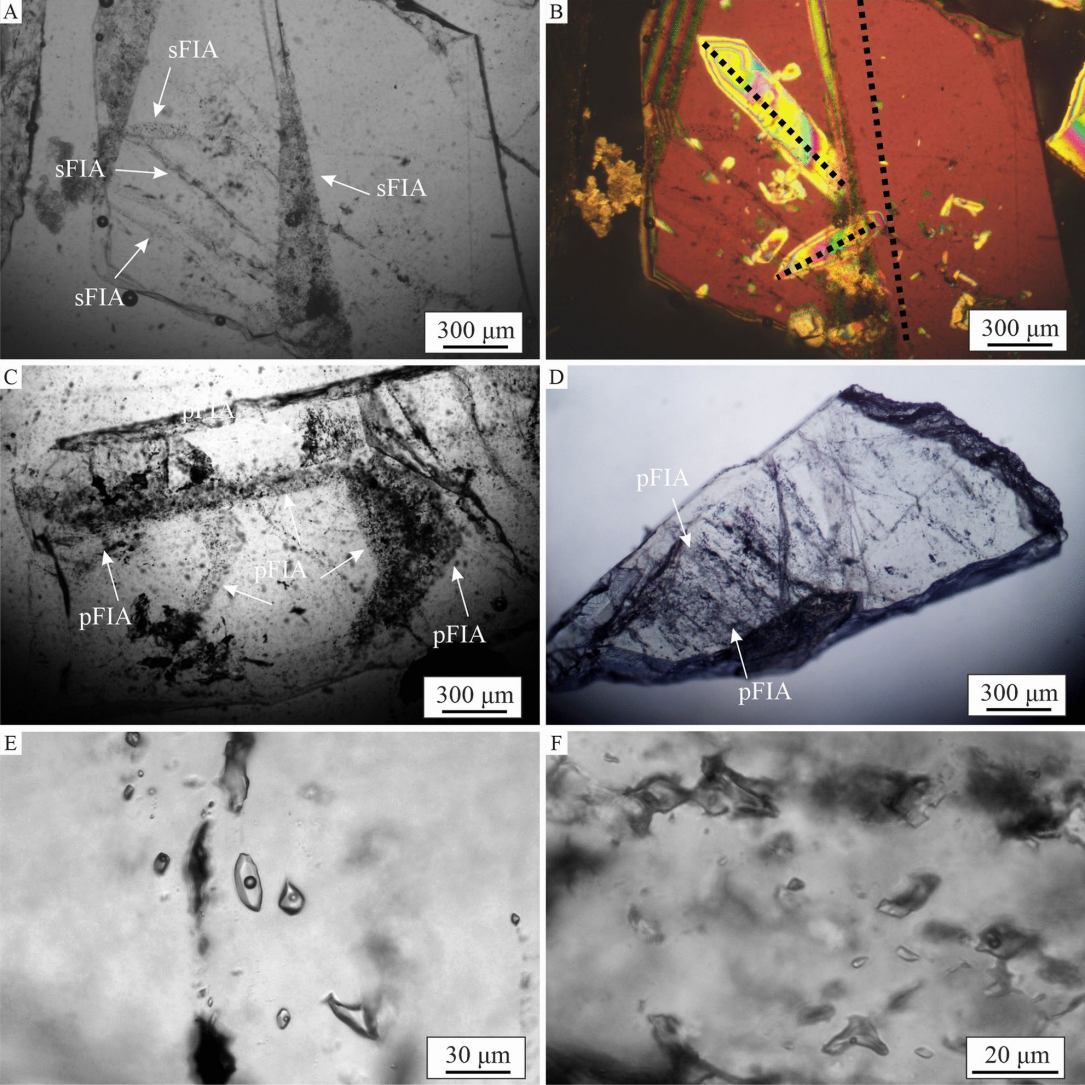
Microphotographs showing fluid inclusions assemblages in quartz (**A**, **B**, **C**, **E**, **F**) and calcite (**D**). Arrows show sFIA—secondary fluid inclusions assembly and pFIA – primary fluid inclusions assembly. The black dotted lines indicate the crystal axes.

Quartz and calcite crystals are commonly of euhedral or subhedral shape. Locally they are clean and transparent under transmitted light but mostly they host densely packed fluid inclusion assemblages (FIAs), which make the image blurred. Interestingly, the quartz crystals do not reveal any special features under plane-polarized light (Fig. 7A), while under crossed-polarized light, intergrowths are seen with the older generation of quartz, whose crystallographic axes are inconsistently aligned to the host crystal (Fig. 7B). Locally, in some quartz crystals, sequential overgrowth of older crystals by a new crystal is also well documented (Fig. 7C). In both cases, the boundaries of the older crystals are marked by fluid inclusions of primary genesis. In rare cases, primary FIAs occupy other, small parts of the crystals and they are mostly small (up to 5 µm in size) with an irregular shape.

**Fig. 8:**
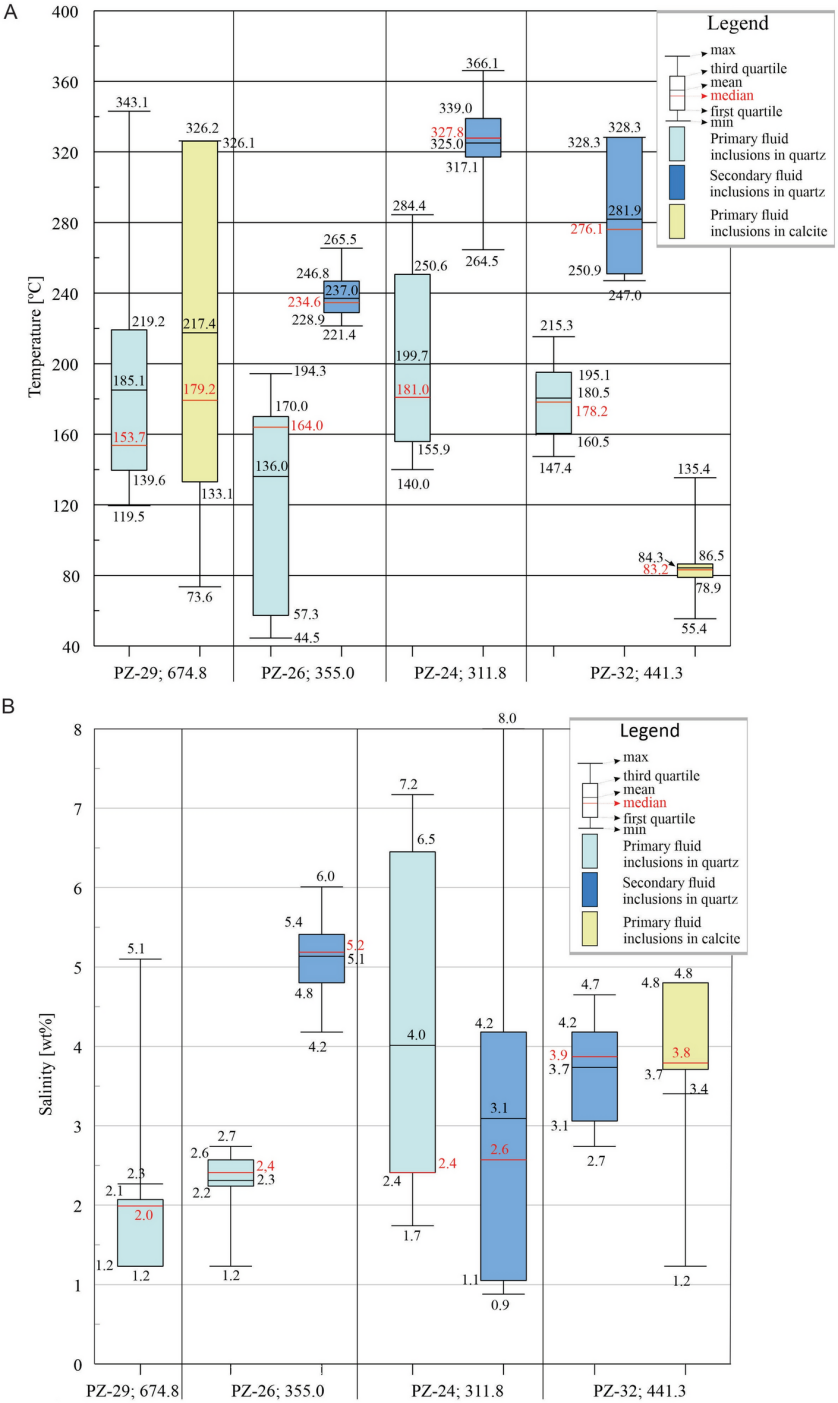
(**A**) Boxplots of homogenization temperatures of fluid inclusions hosted by late-stage quartz and calcite. (**B**) Boxplots of salinities of fluid inclusions hosted by late-stage quartz and calcite.

Most of the quartz crystals have multiple secondary FIAs (Fig. 7A, E). They are most commonly of irregular to almost negative crystal shapes with sizes from about 1 µm to 20 µm. The primary FIAs are typically tiny (up to 15 µm) and irregular in shape (Fig. 7F). Microthermometric measurements show that most fluid inclusions homogenize to a liquid phase. Only in the sample Pz-24, there were two cases, where secondary inclusions homogenized to the gas phase (Th = 327.5 °C, 328.5 °C), while other inclusions found nearby homogenized to the liquid phase (Th = 333.2, 331.8 °C). Such observations may suggest a discrete boiling event. In general, the microthermometric measurements revealed lower homogenization temperature (Th) values for primary FIAs than for secondary FIAs (Fig. 8). For the primary FIAs, the range of the Th is wide (44.5–343.1 °C), and their highest values coincide with the lower limit of Th range obtained for the secondary FIAs. In most samples, the median is lower than the mean value, which means that there are high extreme values in the whole population (Fig. 8A). The exception are the primary FIAs in the Pz-26 sample, where the median is significantly higher than the mean value.

Microthermometric measurements for calcite were only feasible for two samples (Pz-29, and Pz-32). For Pz-29, the range of Th values for calcite fluid inclusions is large and coincides with the range for primary inclusions in quartz (Fig. 8A). In the sample Pz-32, the Th range is low, and half of the measurements are found in the distinctly narrow range of 78.9–86.5 °C (Fig. 8A). Estimated salinities of fluid inclusions in all samples are rather small, especially compared to the data of Karwowski^32^, and do not exceed 8wt% eqNaCl (Fig. 8B). In the Pz-24, the variation is the highest of all samples, but the range of values is quite similar for primary and secondary inclusions (Fig. 8B). Conversely, in the Pz-26 the salinity values for the two types of inclusions differ distinctly and are within relatively narrow limits.

### Pyrite chemistry

Low-temperature, post-hydrothermal pyrite shows a diverse set of elements, either solely structurally-bounded (e.g. As, Au, Co, Cr, Hg, Mn, Ni, Sb, Se, Te, Tl, Ti, and V) or held both in crystal lattice at low concentrations and as micro-scale inclusions (e.g. Ag, Bi, Ce, Cu, In, La, Hf, Pb, Rb, Sn, Zn, Y). The structurally-bonded elements and micro-scale inclusions were identified based on signal fluctuations within an integration interval during LA-ICP-MS analysis. Within the porphyry Myszków Mo–Cu–W deposit, the concentrations of Ag, As, Au, Bi, Co, Ni, Sb, Se, and Te are highly variable, while the concentrations of Cr, Mn, Tl, and Ti are relatively uniform regardless of the deposit zone.

**Fig. 9:**
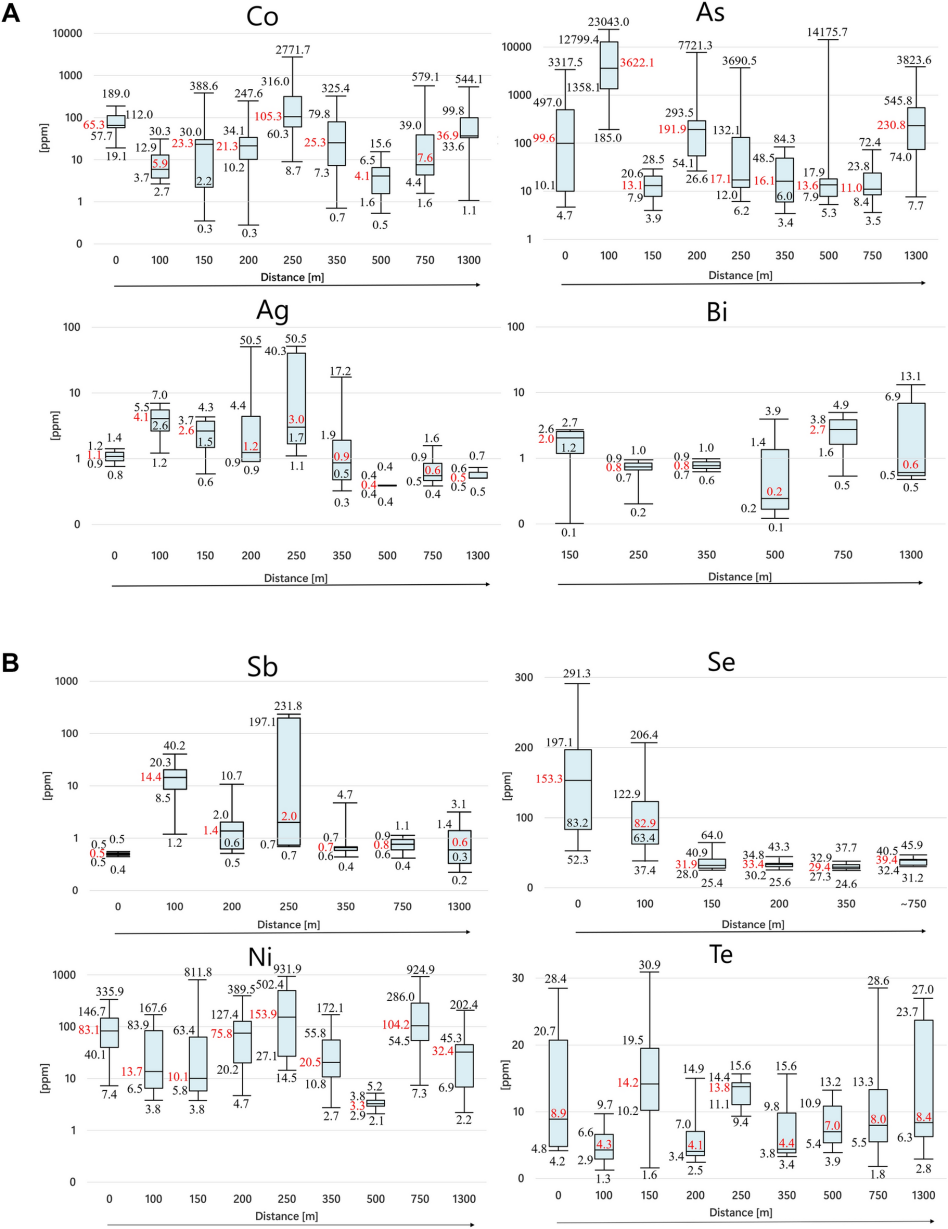
(**A**) Boxplots of the elemental data (Co, As, Ag, Bi) for late-stage pyrite obtained with LA-ICP-MS. (**B**) Boxplots of the elemental data (Sb, Se, Ni, Te) for late-stage pyrite obtained with LA-ICP-MS.

Pyrite from the deposit center shows the highest concentration of structurally-bonded Te (median—mdn. 11.10 ppm), Se (mdn. 63.97 ppm), Bi (mdn. 1.54 ppm), Au (mdn. 17.05 ppm), Sb (mdn. 13.52 ppm), and As (mdn. 184.80 ppm) (Table 5, Fig. 9). However, the high median values of both Sb (13.52 ppm) and As (184.80 ppm) originate from one sample extremely enriched in these elements (Pz-26, 100 m from the deposit center), located in the contact zone between magmatic intrusion and metamorphic wall-rocks. Also, pyrite from the center of the deposit contains the highest budget of In (up to 32.12 ppm), Sn (up to 72.22 ppm), and Cu (up to 5719.74 ppm) related to chalcopyrite inclusions.

**Table 5:** Structurally-bonded trace elements composition [in ppm] of late-stage pyrite from different deposit zones.

	Co	Ni	As	Sb	Te	Se	Tl	Bi	Ag	Ti	Cr	Mn	V
Deposit-zone	23.26	33.02	184.80	13.52	11.10	63.97	0.13	1.54	1.59	10.30	9.94	6.94	0.67
Circum-deposit zone	41.21	59.12	49.22	1.67	5.58	26.80	0.14	0.73	1.68	9.76	12.37	7.33	2.94
Peripheral zone	7.16	45.97	24.96	0.50	7.53	39.35	0.13	0.57	0.50	9.10	10.51	7.59	1.42

Pyrite from the circum-deposit zone shows the highest concentration of structurally-bonded Co (mdn. 41.21 ppm), Ni (mdn. 59.12 ppm), Ag (mdn. 1.68 ppm), and V (mdn. 2.94 ppm) (Table 5, Fig. 9). It also contains the highest budget of Pb (up to 814.63 ppm), probably associated with galena inclusions.

Peripheral zone pyrite differs from other samples mainly in its enrichment of REE-bearing inclusions. It contains increased contents of La (up to 27.32 ppm), Ce (up to 51.25 ppm), Y (up to 1.34 ppm), but also Nb (up to 0.73 ppm) and Hf (up to 2.29) hosted by micro-inclusions.

## Discussion

### Source of sulfur

The overall δ^34^S data of pyrite grains at the porphyry Myszków Mo–Cu–W deposit are between −3.22 and + 10.50 ‰, with most values being over + 5 ‰. Hence, this hydrothermal system appears to be isotopically heavier with respect to the average value of most porphyry-type deposits worldwide, which have δ^34^S from −5 to + 5‰^39,40^. However, heavier S-isotope signature has been previously reported for several porphyry deposits, such as the Sams Creek Au porphyry deposit in New Zealand (+ 5 to + 10‰;^41^), the Sora and Shakhtama Mo porphyry deposits in Siberia (+ 3.8 to + 9.6‰;^42^), and the Diyanqinamu Mo porphyry deposit in Inner Mongolia Autonomous Region (+ 5.2 to + 8.3‰;^43^). The elevated δ^34^S values of the pyrite from the Myszków deposit might be either inherited from the magmatic source or/and the assimilation of isotopically heavy sulfur from the metasedimentary rocks of the Neoproterozoic age intruded by the parent batholith. The porphyry sulfides formed from the H_2_S portion of parental magmatic fluids, after the SO_2_ disproportionation, range from −10 to + 9‰^40^. Moreover, the relatively high δ^34^S values might be consistent with the source of sulfur from mantle-derived rocks, such as basalts and gabbros, that yield δ^34^S values ranging from −11 to + 14‰ [^41^ and the literature therein]. However, the magmatism in the Kraków-Lubliniec Fault Zone was related to two different sources: enriched mantle and primitive crust^44^. Another probable scenario involves the interaction between magma and sulfur-bearing country rocks as sulfur is susceptible to mobilization^41^. According to the authors’ best knowledge, there is a lack of data regarding the isotopic composition of Neoproterozoic flysch from the Małopolska Block. However, some worldwide data indicate the presence of a heavy sulfur composition of some sedimentary pyrites of the Neoproterozoic age (up to + 37.60 ‰, e.g.^45^.).

### Temporal and lateral zonation of δ^34^S_pyrite_: possible controls and fractionation mechanisms

Several plausible explanations for the differentiation of δ^34^S_pyrite_ composition have been proposed, e.g. sulfate reduction during water–rock interaction processes^20^, magmatic-hydrothermal fluid interaction with biogenic sulfur^19,46^, changes in fluid physicochemical parameters such as increase of *f*O_2_, temperature decrease, pH decrease^39^, isotope fractionation between co-precipitated sulfides^47^, sulfur isotope fractionation during processes of dissolution-reprecipitation^48^, and boiling^49^. Some of these mechanisms will be discussed here for the studied area of the Myszków porphyry-related hydrothermal system.

The temporal zonation of δ^34^S_pyrite_ is defined by the progressive decrease in sulfur composition with the evolution of the ore system. This may be due to the decrease in temperature during the transition from skarn- through the main- up to the late-stage of ore mineralization. Another scenario involves the mixing of original ore-forming fluids with oxidized meteoric waters leading to lower δ^34^S values in sulfides forming at the late stage of the system evolution. Fluid inclusion data obtained by^32^ together with the results presented in this paper support such a hypothesis. The salinity of magmatic and post-magmatic inclusions is high (19–75 wt% eq. NaCl; see^32^), whereas the late post-mineralization event is more likely to be associated with more dilute fluids – up to 8 wt% eq. NaCl. The authors^40^ showed that sulfur isotope fingerprints in ore deposits are controlled by the ratio of SO_2_/H_2_S, which is affected mainly by changing temperature, *f*O_2_ and pH. Earlier,^39^ suggested that fluctuations in *f*O_2_ would affect the isotopic composition of pyrite only when sulfides were in equilibrium with Fe oxides. According to the latest works^50,51^, the higher the oxygen fugacity, the higher the δ^34^S values. In the porphyry Myszków Mo–Cu–W deposit, along with the temperature drop, the systems evolved into a more oxidized environment^17^, when besides sulfides, some sulphates (barite) appeared^29^. Hence, over time and with changes in oxygen fugacity, earlier sulfides with higher δ^34^S values crystallized, while late-stage sulfides formed lighter species due to sulfate-sulfide fractionation. Moreover, local events such as phase separation, the interaction of magmatic-hydrothermal fluid with more oxidized country rocks, fluid mixing with a more reduced sulfur source, and a distinct fluid evolution pathway might lead to temporal ^34^S enrichment or depletion, showing incidental values being off-trend (Table 2).

Both lateral and vertical zonation of δ^34^S_pyrite_ is well-developed only for the late-stage mineralization event, for which it shifts towards negative values (Figs. 5A,B and 6A,B). One of the crucial processes triggering ore formation in magmatic-hydrothermal systems leading to negative δ^34^S values in hydrothermal pyrites could be boiling^52^. However, our results suggest that boiling can affect the isotopic composition of late-stage pyrite only locally. Most of the specimens form almost perfectly euhedral crystals devoid of any inclusions, which may indicate a slow rate of crystallization under constant conditions^53^. However, shifts of δ^34^S_pyrite_ to negative values measured in some inclusion-rich pyrites, namely the sample from the Pz-24 borehole (311.8 m depth, circum-deposit zone), can be interpreted as a result of boiling, which is supported by the fluid inclusions data obtained from the associated quartz. Therefore, boiling was not a critical process for the development of an isotopic halo around the mineralization core, but it could cause local anomalies. It should be also taken into account that, in some cases, boiling may lead to an increase of δ^34^S value in pyrite^54^.

The cooling process itself may provide another way to explain the progressive depletion of δ^34^S_pyrite_ outward from the central heat source towards the surface, however, the isotopic data do not correlate well with lateral temperature variations for quartz (constant Th values; see Table 4), but overlap the temperature decrease for calcite (Table 4). Moreover, the hydrothermal system of the Myszków deposit is complex and the interpretation of fluid inclusion data is quite difficult. The presence of secondary FIAs with higher Th, than that of primary fluid inclusions (Table 4) points to periodic intermittent injection of higher temperature fluids that may also produce core-rim textures of some pyrite crystals. An example is sample Pz-10 (depth 276.6 m), where a higher δ^34^S composition is observed in the pyrite cores than the pyrite rims (Fig. 3C). It was previously suggested that mafic melt injected episodically into the granodioritic pluton during its crystallization as evidenced from the presence of mafic microgranular enclaves within granitoids from the marginal part of the Małopolska Block^55^. On the other hand, the highest homogenization temperatures of primary FIAs, corresponding to Th of secondary FIAs, may result from their unsealing and then sealing under new PTV conditions.

The incorporation of an external, isotopically light sulfur source such as biogenic sulfide has been abandoned as the primary driver for the differentiation of δ^34^S_pyrite_. There is no local source of biogenic sulfur in the porphyry Myszków Mo–Cu–W deposit because the wall rocks hosting the deposit are composed of the Neoproterozoic flysch (Fig. 1) devoid of a significant amount of organic matter. Instead, the presence of the δ^34^S_pyrite_ negative values at the deposit periphery might be attributed to the interaction of ore-forming fluids with hematite-altered wall rocks^21^.

### Lateral zonation of δ^34^S_pyrite_: a new tool for exploration?

The well-developed zonation of δ^34^S_sulfide_values for late-stage pyrite raises the question of whether it can be used as another vector toward the mineralization center in porphyry systems. An isotopic halo around the mineralization core was found to extend laterally for a distance of up to 3 km. Hence, it could potentially be used as an exploratory guide for undercover exploration within the whole Kraków-Lubliniec belt, which constitutes the larger Trans-European Composite Tectonic Suture Zone – one of the promising areas for discovering new porphyry-type deposits to supply critical metals to the European Union^56,57^. Detection of an increasing sulfur isotopic composition in late-stage pyrite encountered during drilling programs may indicate that the mineralized core is being approached. However, this zonation pattern appears to be province specific. Most of the porphyry deposits have sulfides with strongly negative sulfur isotopic compositions in their cores and with near-zero values on their peripheries^58^. However, quite recently a similar isotopic signature was observed in the porphyry Altar Cu-Au deposit (Argentina) with more δ^34^S negative values in the periphery of the main Cu zone^59^.

Furthermore, it seems that δ^34^S_pyrite_ composition is not merely a function of distance but may also be affected by other factors such as depth, boiling conditions, and pulses of high-temperature fluids. As a result, the vertical pattern of δ^34^S_pyrite_ (progressive depletion in heavy sulfur toward the surface in the circum-deposit zone and peripherical zone; see Table 3) might be also used for prospecting. The presence of pyrite, which shows evidence of boiling (i.e. inclusion-rich textures, shifts toward negative δ^34^S_pyrite_ values) in the shallow environment might suggest the possibility of significant mineralization nearby. Hence, with sufficient sampling density, pyrite isotopic signature might become a mature tool for tracing fluid flow pathways. The *in-situ* method of analysis is preferable to reveal core-rim textures of pyrite and related changes in sulfur isotope signature.

### Geochemical zonation of late-stage pyrite: new pathfinders to ore

**Fig. 10:**
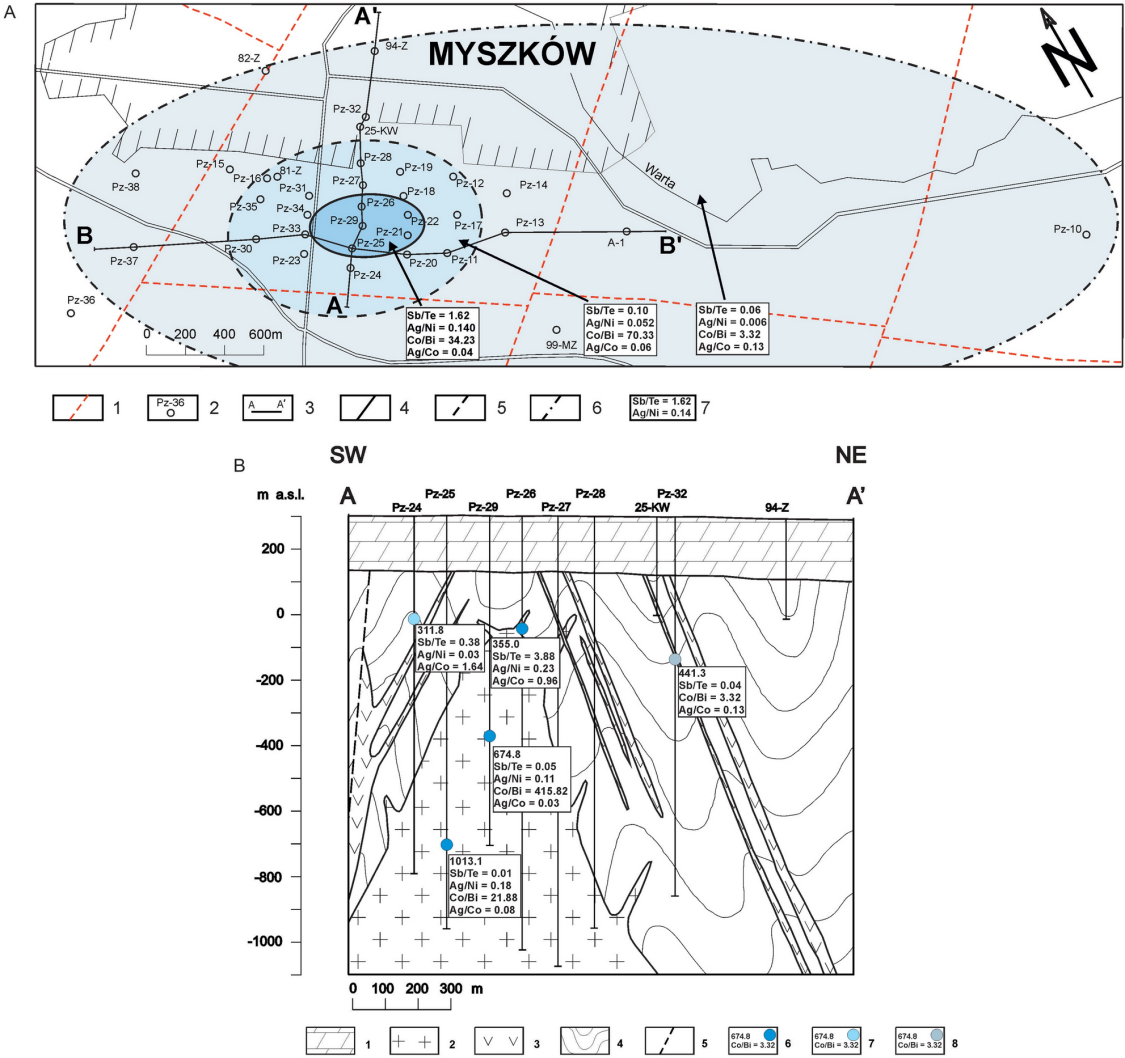
(**A**) Map of the Myszków Mo–Cu–W deposit with the elemental ratios of late-stage pyrite. 1—faults, 2—boreholes, 3—geological cross-section lines, 4—limit of deposit zone, 5—limit of the circum-deposit zone, 6—limit of deposit peripheral zone, 7—data with selected elemental ratios of pyrite from the central deposit zone, 8—data with selected elemental ratios of pyrite from the circum-deposit zone, 9—data with selected elemental ratios of pyrite from the peripheral zone. (**B**) NW–SE cross-section through the Myszków deposit. 1—Triassic sediments, 2—granitoids, 3—dacitoids, 4—metamorphosed clastic rocks of Vendian-Early Cambrian, 5—faults, 6—investigated points with late-stage pyrite from the central deposit zone (depth, elemental ratios), 7—investigated points with late-stage pyrite from the circum-deposit zone (depth, elemental ratios), 8—investigated points with late-stage pyrite from the peripheral zone (depth, elemental ratios).

When considering the bulk rock geochemistry of the porphyry Myszków Mo–Cu–W deposit as a function of distance-to-center, a significant increase in Sb and Tl content is observed for late-stage mineralization event^60^. In general, the circum-deposit zone is characterized by increased concentrations of Hg and Au, while the peripheral zone is associated with a positive anomaly of Bi and Te^60^. Pyrite, as a mineral that retains the substantial characteristics of its ore, is believed to follow that trajectory. Recently, promising results published by^16^ hinted at a systematic behavior of some trace elements in pyrite from a porphyry-skarn environment. Xiao et al.^16^ noted that pyrite-hosted Co and Ni increase toward the center of mineralization, while As, Sb, Pb, Ag, and Bi are enriched in more distal samples. In our study, a clear trend is observed only for late-stage pyrite-hosted As, Sb, and Bi. Their content distinctly increases towards the center of mineralization. Similarly, Co and Ag rapidly decrease in the peripheral zone (Table 5). The elemental ratios of Sb/Te, Co/Bi, Ag/Ni, and Ag/Co calculated for late, post-hydrothermal pyrite appear to be the most promising vector proxies for predicting the probable direction to the mineralized center (Table 6, Fig. 10). There is a gradual decrease in the elemental ratios of Sb/Te, Co/Bi, and Ag/Ni with distance from the deposit core, through the circum-deposit, to the distal zone. On the other hand, the Ag/Co ratio shows the opposite trend, with values increasing towards the periphery of the deposit. Hence, in addition to being an important petrogenetic indicator^61–63^ pyrite could be also considered as a porphyry vectoring mineral.

**Table 6:** Proposed geochemical pyrite-based proxies towards mineralization center.

	Co/Ni	Se/Te	Co/As	Sb/Te	Co/Bi	Ag/Ni	Ag/Co
Deposit-zone	0.54	6.12	0.22	1.62	34.23	0.140	0.04
Circum-deposit zone	0.61	4.26	0.71	0.10	70.33	0.052	0.06
Peripheral zone	0.35	4.16	0.46	0.06	3.32	0.006	0.13

According to^64^ the temperature-dependent elements in pyrite include As, Bi, Sb, and Se; while Co and Ni are less sensitive to this factor. Interestingly, the systematic decrease of As, Bi, Sb, and Se together with rapid drop of Co content towards the periphery of the porphyry Myszków Mo–Cu–W deposit is consistent with the decrease of δ^34^S values for late-stage pyrite. Bismuth, Co, and Se are considered as indicators of higher temperature of pyrite formation, while As and Sb are rather related to lower conditions^65^, being however, also sensitive to redox state^66^. Depletion in As and Sb content in pyrite might point to the more oxidized fluids from which late-stage mineralization precipitated. Hence, integration of geochemical and isotopic data excludes simple cooling as a sufficient explanation for sulfur isotopic fractionation observed for late-stage pyrite. Additional driver could be related to the changes in *f*O_2_ resulted from increasing contribution of meteoric waters and/or fluid interaction with hematite-bearing wall-rocks at the deposit periphery.

## Conclusions

The work was aimed to develop an isotopic-based approach to vectoring toward the mineralization center in the porphyry Cu system using the porphyry Myszków Mo–Cu–W deposit (the Krakow-Lubliniec Fault Zone, Central Poland) as a case study. The δ^34^S_pyrite_ values define well-developed zoning across the mineralization stages of the porphyry Myszków Mo–Cu–W deposit, probably resulting from the temperature decrease and/or mixing of initial ore-forming fluids with meteoric waters coupled with fluctuations of oxygen fugacity and sulfate-sulfide fractionation mechanism. Additionally, there is a gradual decrease in δ^34^S of late-stage pyrite outwards from the deposit core to the peripheral zone. Thus, pyrite isotopic composition has the potential to be used as another tool to assist future drilling programs in porphyry provinces. However, using pyrite isotopic composition as a vector toward the core of the mineralized system requires careful consideration due to the local processes that may have influenced pyrite isotopic signatures, e.g. boiling, dissolution-reprecipitation, and injection of higher-temperature fluids. Data supporting this assumption can be obtained from the pyrite trace geochemistry, especially the contents of As, Sb, Bi, Co, Ag, and elemental ratios of Sb/Te, Co/Bi, Ag/Ni, Ag/Co, which seem to be the most promising tool for ore prospecting in the Mo–Cu–W Myszków deposit and elsewhere.

### Sampling and analytical methods

The 29 samples of porphyry-style pyrite representing various mineralization stages and different zones within the Myszków Mo–Cu–W deposit were analyzed for sulfur isotopic compositions. Specifically, the three generations of pyrite, i.e. (1) early, skarn forming; (2) main stage, hydrothermal, and (3) late, post-hydrothermal, all originating from the central core deposit (boreholes no Pz-25, Pz-26, Pz-29), circum-deposit (boreholes no Pz-16, Pz-30, Pz-33), and peripheral (boreholes no Pz-15, Pz-32, Pz-37) zones were investigated in this work. The sulfur isotope analyses were performed by using an ion microprobe equipment – SHRIMP IIe/MC, that allows avoiding of other mineral micro-inclusions or microfractures within pyrite crystals.

Additionally, to discuss the sulfur fractionation mechanism and discriminate specific causes for isotopic variations within the late, post-hydrothermal pyrite, the fluid inclusion data from associated late-stage quartz and calcite were made. The fluid inclusions analyses were carried out on samples collected from veins and small geodes from Pz-26, Pz-29 (central, ore deposit zone) Pz-24 (circum-deposit zone), and Pz-32 boreholes (peripheral zone) located along the SW-NE cross section. Finally, the geochemical analysis via LA-ICP-MS was provided for the late-stage pyrite to establish new pathfinders to ore. The samples from drill holes no Pz-29, Pz-26, Pz-25 (central, ore deposit zone), Pz-24, Pz-18, Pz-19 (circum-deposit zone), and Pz-32, Pz-15, Pz-37 boreholes (peripheral zone) were selected for LA-ICP-MS study.

*SHRIMP IIe/MC* Single pyrite grains were handpicked from rock fragments and mounted in epoxy resin with reference pyrite Rutan (δ^34^S =  + 1.20 ± 0.10‰ VCDT) and Balmat (δ^34^S = 15.37 ± 0.62‰ VCDT)^67^ polished and coated with gold for sensitive high-resolution ion microprobe (SHRIMP IIe/MC) analysis conducted in Polish Geological Institute-National Research Institute (PGI-NRI) in Warsaw, Poland.

The mounts were cut to the appropriate thickness, and pre-polished on an automatic polishing machine with polishing solutions of successively 9, 3, 1, and 0.25 µm grain size. Prior to analysis on the SHRIMP ion microprobe, images of the mount surface were taken with a reflected light optical microscope and with a scanning electron microscope (BSE detector) to select inclusion free spots. Then the surface of the mount was coated with ultra-pure gold with a thickness of 25 nm.

Before each measurement, the sample surface was cleaned by the primary beam for 120 s to form a raster with a diameter larger than the measurement spot to remove gold and other contamination. For the measurements, 3 nA of the primary Cs + beam was focused into a spot with a diameter of ~ 23 μm. Secondary ions were pulled from the surface using a low potential of ~ 800 V extraction voltage. The total accelerating voltage of the secondary beam was about 10 kV while the primary beam accelerating voltage was about 15 kV. The resolution (M/dM) was about 2000 measured at 10% peak height. Measurement of sulfur isotopes included two sets of five 32S and 34S mass scans simultaneously measured on Faraday Cups. The Faraday Cups electrometer resistors used to measure the 32S and 34S masses were set at 1011 Ω. The total time of measurement of one point was about 6 min. The results were reduced using the POXY MC v2.9 program.

*Fluid inclusions petrography and microthermometry* The fluid inclusions analysis was performed to support the sulfur isotope study and to verify the assignment to a specific pyrite generation. Microthermometric measurements were conducted with a Linkam THMSG600 Geology Heating and Freezing Stage compatible with the T96 temperature control system manufactured by Linkam. The stage was mounted on an Olympus BX53 microscope using 5x, 10x, 20x, 50x, and 100 × objectives equipped with a UC90 Olympus camera. It was calibrated using pure CO_2_ synthetic inclusions (Tm =  −56.6 °C) and the known homogenization temperature of pure H_2_O inclusions. A heating-freezing rate of 2–5 °C/min with an accuracy of 0.1 °C was applied.

FIA types (primary, secondary and pseudosecondary) have been distinguished depending on their relationships to crystallographic axes and mode of occurrence^36–38^. Primary FIAs are arranged along the crystal growth surface, forming bands with variable inclusion packing density (Fig. 7F). Secondary FIAs form linearly arranged inclusions with longer axes, marking fracture surfaces in the crystals (Fig. 7E). Very often, the distinction between individual types was ambiguous due to the large number of inclusions making microscopic observations difficult, and the overlapping and/or intersection of FIAs of different origins.

*LA-ICP-MS* Trace element concentrations in pyrite were measured using a PerkinElmer ELAN DRC-e ICP mass spectrometer combined with a New Wave UP193FX laser ablation system, equipped with ATLEX-LR ArF excimer laser at the Geological Institute, Bulgarian Academy of Sciences (Sofia, Bulgaria). To maximize sensitivity, the ICP-MS was optimized daily for the ThO/Th oxide production rate (0.5%). The analyses were performed with 35 μm beam spots and a repetition rate of 3–6 Hz (depending on the thickness of the polished thin section) with a homogeneous energy density on the analyzed minerals and standards of 6.0–6.6 J/cm^2^. The nebulizer gas flow rate was 0.74–0.76 L/min, while auxiliary and make-up gas (helium) flow rates were 0.90 L/min and 0.92 L/min, respectively. The analysis time was 70–90 s (background: 40 s, laser-on the sample ~ 30–50 s, due to thinness of the polished thin section). The acquisition dwell times were set to 0.02 s for ^75^As, ^107^Ag, ^121^Sb, ^202^Hg, ^205^Tl; to 0.03 s for ^82^Se, ^125^Te; to 0.04 s for ^197^Au, and 0.01 s for all other monitored isotope masses—^34^S, ^49^Ti, ^51^ V, ^53^Cr, ^55^Mn, ^57^Fe, ^59^Co, ^60^Ni, ^65^Cu, ^66^Zn, ^71^ Ga, ^74^Ge, ^95^Mo, ^106^Pd, ^111^Cd, ^115^In, ^118^Sn, ^181^Ta, ^182^W, ^208^Pb, and ^209^Bi. The analyses were done on previously measured spots by electron microprobe. Repeated external standardization by analyzing NIST SRM 610 glass standard and the USGS Mass 1 sulfide standard allows linear drift correction of the mass spectrometer and provides relative concentrations of elements. These concentrations are calculated into true values by using Fe content measured by electron microprobe as internal standard and the Matlab®-based SILLS software Version 1.3.2^68^. During data reduction, peak-shaped fluctuations of the intensity signal of some isotopes were excluded where it was possible to minimize the influence of other minerals on the chemical composition of pyrite.

## Data Availability

Raw data are available at: 10.58032/AGH/UGHHRL.
